# Colonic Motility and Jejunal Vagal Afferent Firing Rates Are Decreased in Aged Adult Male Mice and Can Be Restored by an Aminosterol

**DOI:** 10.3389/fnins.2019.00955

**Published:** 2019-09-10

**Authors:** Christine L. West, Jessica Y. Amin, Sohana Farhin, Andrew M. Stanisz, Yu-Kang Mao, Wolfgang A. Kunze

**Affiliations:** ^1^St. Joseph’s Healthcare, The Brain-Body Institute, McMaster University, Hamilton, ON, Canada; ^2^Department of Biology, McMaster University, Hamilton, ON, Canada; ^3^Department of Psychiatry and Behavioural Neurosciences, McMaster University, Hamilton, ON, Canada

**Keywords:** aging, vagal afferent, motility, constipation, squalamine

## Abstract

There is a general decline in gastrointestinal function in old age including decreased intestinal motility, sensory signaling, and afferent sensitivity. There is also increased prevalence of significant constipation in aged populations. We hypothesized this may be linked to reduced colonic motility and alterations in vagal-gut-brain sensory signaling. Using *in vitro* preparations from young (3 months) and old (18–24 months) male CD1 mice we report functional age-related differences in colonic motility and jejunal mesenteric afferent firing. Furthermore, we tested the effect of the aminosterol squalamine on colonic motility and jejunal vagal firing rate. Old mice had significantly reduced velocity of colonic migrating motor complexes (MMC) by 27% compared to young mice (*p* = 0.0161). Intraluminal squalamine increased colonic MMC velocity by 31% in old mice (*p* = 0.0150), which also had significantly reduced mesenteric afferent single-unit firing rates from the jejunum by 51% (*p* < 0.0001). The jejunal vagal afferent firing rate was reduced in aged mice by 62% (*p* = 0.0004). While the time to peak response to squalamine was longer in old mice compared to young mice (18.82 ± 1.37 min vs. 12.95 ± 0.99 min; *p* = 0.0182), it significantly increased vagal afferent firing rate by 36 and 56% in young and old mice, respectively (*p* = 0.0006, *p* = 0.0013). Our results show for the first time that the jejunal vagal afferent firing rate is reduced in aged-mice. They also suggest that there is translational potential for the therapeutic use of squalamine in the treatment of age-related constipation and dysmotility.

## Introduction

Old age is associated with increased incidence of chronic constipation, which increases in prevalence with age (Higgins and Johanson, 2004; De Giorgio et al., 2015; Ranson and Saffrey, 2015). In addition, aging is also associated with behavioral depression (Malatynska et al., 2012). Age-related changes in the gut and gut to brain nervous signaling via the vagus nerve may underlie many of these problems in the geriatric age group, although psychosocial and economic factors may play a role (Strawbridge et al., 1996). Old animals show delayed gastric emptying, slowed colonic transit and reduced fecal output (Smits and Lefebvre, 1996). For example, 2-year-old mice deliver fewer fecal pellets than 3-month-old ones, and when epoxy coated pellets are introduced into the colon of old mice they move with decreased velocity in the oral to anal direction (Patel et al., 2012).

Whether age-related changes in intestinal propulsion are due to alterations of smooth muscle function or cells that coordinate or pace contractions such as neurons or interstitial cells of Cajal (ICC), is unclear (Saffrey, 2014). However, while reductions in the number of ICC and enteric glial cells have been reported in the aged gut (Saffrey, 2014) the enteric nervous system (ENS) is also vulnerable to age-related damage. Generation and propagation of colonic migrating motor complexes (CMMCs) in mice are generated by activity of the ENS (Fida et al., 1997; Roberts et al., 2007; Spencer et al., 2018), recorded *in vitro* (Wang et al., 2010a, b; Wu et al., 2013) and are absent if the ENS is missing or destroyed as in Hirschsprung’s or Chagas’ diseases (Furness, 2006, p. 157). Indeed peristalsis, but not ICC dependent slow wave related contractions, is abolished by tetrodotoxin (Wu et al., 2013; Delungahawatta et al., 2017). In fact, neurogenic migrating motor complexes still occur in mutant mice lacking pacemaker-type ICC and slow waves in the small intestine (Spencer et al., 2003).

The myenteric plexus of the ENS is essential for normal MMCs to occur in the colon (Fida et al., 1997; Roberts et al., 2007; Wang et al., 2010b; Spencer et al., 2016, 2018). Intrinsic primary afferent neurons (IPANs) represent the class of myenteric neurons most affected by degenerative changes in old age (Wade, 2002; Wade and Cowen, 2004) and MMCs are absent if they are selectively silenced (Howe et al., 2006). However, the ENS appears to be more susceptible to age-related degeneration than other nervous systems (Saffrey, 2013). While some animal studies suggest that there may be reductions in the number of myenteric neurons in old age (El-Salhy et al., 1999; Phillips et al., 2004; Phillips and Powley, 2007; Zanesco and Souza, 2011), it is probable that myenteric neuron numbers are actually maintained, but an increasing proportion show structural degenerative changes with increasing old age (Gamage et al., 2013; Saffrey, 2013).

We are not aware of extant data on age-related functional changes in vagal nerves, but vagal afferents in aged rats have swollen varicosities in fibers innervating the myenteric plexus, smooth muscle and mucosa (Phillips and Powley, 2007). There is no information available whether there is an actual decrease in the number of vagal fiber endings supplying the myenteric plexus. However, dystrophic changes including dilations and swellings of the intraganglionic laminar endings (IGLEs) in the NIH Fisher 344 rat model of aging have been described and the extent of the terminal arbors is also reduced compared to young rats (Phillips et al., 2010). A previous study showed that aged mice had attenuated colonic and jejunal afferent mechanosensitivity and suggested that the loss or decrease of this sensory innervation or sensitivity may be linked to the reduced awareness of constipation in the elderly (Keating et al., 2015).

In the present paper we report the effects of old age on colon motility and jejunal vagal afferent firing using *in vitro* preparations from male CD1 mice. Squalamine is a prokinetic aminosterol originally synthesized by the liver of the dogfish shark (Zasloff et al., 2011), and it has previously been shown to stimulate colonic motility in a 1-year-old mouse and loperamide model (Kunze et al., 2014). Here we explore in detail the effects of old age (2-year) on colon motility and constitutive vagal afferent firing rates from the jejunum, and whether these functions might be restored to youthful levels by the aminosterol squalamine.

## Materials and Methods

### Animals

Young (3 months) and old (18–24 months; retired breeder) male CD-1 mice from Charles River Laboratories (Quebec, Canada) were used for all portions of the study. Experiments were performed *in vitro* following cervical dislocation in accordance with the Animal Research Ethics Board (AREB) of McMaster University (permit 16-08-30). Mice were housed on a 12-hour light/dark cycle, food and water were provided *ad libitum*, and mice were allowed 1-week acclimatization following arrival. In view of the current debate about sex differences in mammalian nervous systems (O’Connor and Cryan, 2014), we plan to conduct future studies with female CD1 mice when these become available after an 18–24-month aging period.

### Colonic Motility

The colonic motility recordings were performed as described previously (West et al., 2017). The whole colon was extracted from young (3 months) and old (18–24 months) mice, flushed with Krebs, and cannulated with silicon tubing at the oral and anal ends within a heated, Krebs-filled tissue flotation bath. Krebs was prepared with the following concentrations (mmol L^–1^): 118 NaCl, 4.8 KCl, 25 NaHCO_3_, 1.0 NaH_2_PO_4_, 1.2 MgSO_4_, 11.1 glucose, and 2.5 CaCl_2__,_ bubbled with carbogen gas (95% O_2_ and 5% CO_2_) and heated to 37°C (Wu et al., 2013). The inflow (oral end) and outflow (anal end) tubes were adjusted in height to create an intraluminal pressure difference of 2–3 hPa during perfusion of the lumen. Gravity-evoked contractions were recorded by a Microsoft Lifecam 3000 web camera positioned 8 cm above the tissue. The serosal compartment of the bath was constantly perfused with fresh oxygenated Krebs. Squalamine (10 μM) was applied luminally by opening and closing the stopcocks of the Mariotte tubes at the oral end [see Figure in Wu et al. (2013)].

Intestinal contractions were recorded during a 20 min Krebs control and 20 min treatment period. Spatiotemporal diameter maps were developed from the motility video recordings as described previously (Wu et al., 2013; West et al., 2017). Alternating dark and light bands indicate contraction and relaxation along the gut representing migrating motor complexes (MMCs). MMC velocity was measured as the slope of the large dark contractions. MMC frequencies were determined by measuring the number of MMCs over a given time interval and amplitude was measured as a function of the gut diameter at peak contractions.

### Mesenteric Nerve Recordings

The mesenteric nerve bundle is a mixed nerve containing populations of vagal and spinal fibers (Perez-Burgos et al., 2013). Jejunal mesenteric nerve recordings were performed as described previously (Perez-Burgos et al., 2013). A 2–3 cm segment of mouse jejunum with attached mesentery was excised and mounted on an agar-coated petri dish filled with oxygenated Krebs buffer and nicardipine (3 μM) to paralyze smooth muscle. The oral and anal ends of the tissue were cannulated with silicon tubing and the luminal contents were flushed using Krebs. The remaining mesentery was pinned out and the mesenteric nerve bundle was dissected using fine-tipped forceps. The petri dish was then mounted on an inverted microscope and the nerve bundle was sucked onto with a glass micropipette attached to a microelectrode. The nerve preparation was continuously perfused with fresh oxygenated Krebs in the serosal compartment using a pump. Multi-unit electrical activity was recorded using a Multi-Clamp 700B amplifier and Digidata 1440A signal converter (Perez-Burgos et al., 2013). Control periods were recorded for 15–30 min during luminal Krebs perfusion. Intraluminal squalamine (10 μM) was perfused following the control (Krebs) for a duration of 30 min. Cholecystokinin (CCK) was applied 10 min after the cessation of treatment and Krebs washout to allow for identification of vagal fibers during *post hoc* computer analysis as vagal fibers respond potently to CCK, while spinal fibers do not (Richards et al., 1996; Hillsley and Grundy, 1998). Lastly, 5HT_3_ agonist was applied as it activates a small population of vagal afferent fibers not activated by CCK (Hillsley and Grundy, 1998) and a 37 hPa distention for 1 min tested response to painful distention or a high-threshold stimulus (Perez-Burgos et al., 2015).

Multi-unit electrical activity was analyzed for single-unit activity using principle component analysis (PCA) and spike waveform analysis in the DataView program (Heitler, 2007). Each single-unit fiber has a unique action potential spike that is distinguished from other single fibers by its shape, size and duration (Heitler, 2007; Perez-Burgos et al., 2013). Once sorted into single-units, vagal fibers were identified by response to CCK, as described previously. Single-unit vagal activity was gated for control and treatment periods and the mean interval between spike firing (the inverse of firing frequency) was measured. Decreases in the interspike interval are described as increased afferent firing rate and vice versa.

### Statistics

Researchers were not blinded to experimental groups (young vs. old mice). Percent difference was calculated by (treatment-control)/control for paired before and after treatment or (old-young)/young for age comparisons. Data are presented as mean ± SEM. *N* represents number of mice. Where multiple afferent fibers are measured from one animal, *N* is represented as *N* = # of mice (# of fibers). Statistical comparisons were performed using paired or unpaired, two-tailed *t*-tests using GraphPad Prism software (Version 7.0). Any outliers were identified or removed using Grubbs’ test (α = 0.05) or the ROUT Method (*Q* = 1%).

## Results

### Colonic Contractile Motility Is Reduced in Aged Mice

**FIGURE 1:**
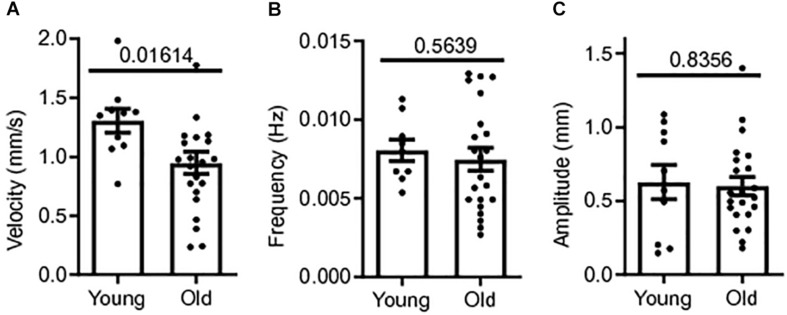
Colonic contractile motility is reduced in aged mice. **(A)** Sample MMC velocity was decreased in old mice (*N* = 22) compared to young mice controls (*N* = 10). **(B)** Sample MMC frequency in old mice compared to young mice controls. **(C)** Sample MMC amplitude in old mice compared to young mice controls. Data represented as mean with SEM, *t*-test unpaired, two-tailed, with Welch’s correction.

Colonic contractile motility was assessed in a total of 32 male CD1 mice; 22 old mice (18–24+ months) and 10 young mice (3 months). The whole length of the colon was excised and the colonic MMCs were video recorded in our gut motility apparatus during Krebs luminal perfusion for later measurements of MMC velocity, frequency and amplitude. Means for MMC velocity in old CD-1 mice during Krebs control were significantly reduced compared to young mice controls. Mean MMC velocity in old mice controls was 0.948 ± 0.09 mm/s, 27% slower than young mice controls, 1.31 ± 0.10 mm/s (*p* = 0.0161) (Figure 1A). MMC frequency and amplitude were not significantly affected by age. Mean frequency was 7.2% smaller in old mice controls (0.007 ± 0.001 Hz) compared to young mice controls, 0.008 ± 0.001 Hz (*p* = 0.5639) (Figure 1B). Mean MMC amplitude in old mice was 0.601 ± 0.062 cm, 4.4% smaller than for young mice, 0.628 ± 0.116 cm (*p* = 0.8356) (Figure 1C). Based on these findings there was a reduction in contractility with age in the colon, with the greatest effect being a reduction in MMC velocity.

### Squalamine Restores Colon Motility in Aged Mice

**FIGURE 2:**
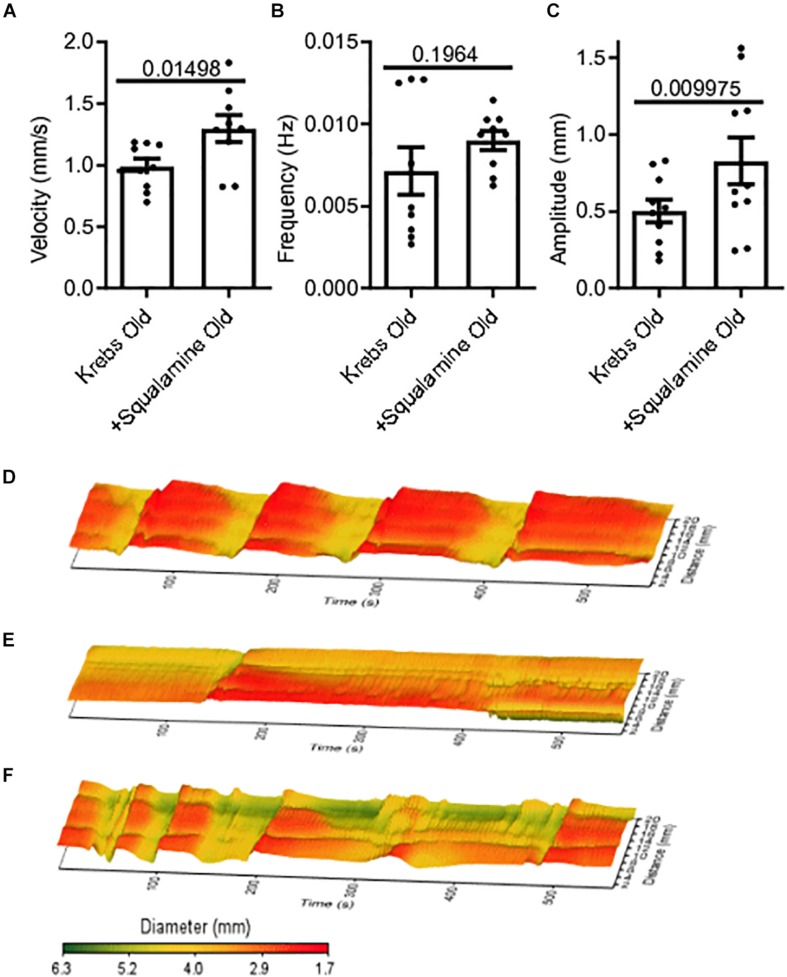
Reduced colonic motor activity could be partially restored by intraluminal application of the aminosterol squalamine. **(A)** Intraluminal squalamine (10 μM) increased sample colonic MMC velocity in aged mice (*N* = 10). **(B)** Intraluminal squalamine and colonic MMC frequency in aged mice. **(C)** Intraluminal squalamine increased colonic MMC amplitude in aged mice. 3D spatiotemporal heat maps display MMC contractions over time (*x*-axis) and distance (*y*-axis). Areas of contraction or small diameter are red, while areas of relaxation or larger diameter are yellow to green for **(D)** a young mouse during Krebs control, **(E)** an old mouse during Krebs control, and **(F)** an old mouse after intraluminal treatment with squalamine. Data represented as mean with SEM, paired *t*-tests, two-tailed.

Squalamine (10 μM) was added *in vitro* to the lumen of colon segments taken from old mice (*N* = 9), following a 20-minute period of control recording with only Krebs in the lumen. Changes to MMC parameters before and after adding squalamine were measured from spatiotemporal maps. Intraluminal squalamine significantly increased mean velocity by 31% from 0.99 ± 0.06 mm/s during Krebs control to 1.3 ± 0.11 mm/s (*p* = 0.0150) (Figure 2A). Intraluminal squalamine increased frequency 26% from 0.007 ± 0.001 to 0.009 ± 0.001 Hz (*p* = 0.1964), but not within statistical significance (Figure 2B). Amplitude was significantly increased 65% from 0.50 ± 0.07 to 0.83 ± 0.15 mm (*p* = 0.0100) (Figure 2C). Spatiotemporal heat maps of MMCs demonstrate a loss of contractile motility and regularity in old (Figure 2E) compared to a young mouse (Figure 2D). Propulsive contractility was restored in the old mice following application of intraluminal squalamine (Figure 2F).

### Single-Unit Firing From the Mesenteric Afferent Nerve Bundle Is Reduced in Aged Mice

**FIGURE 3:**
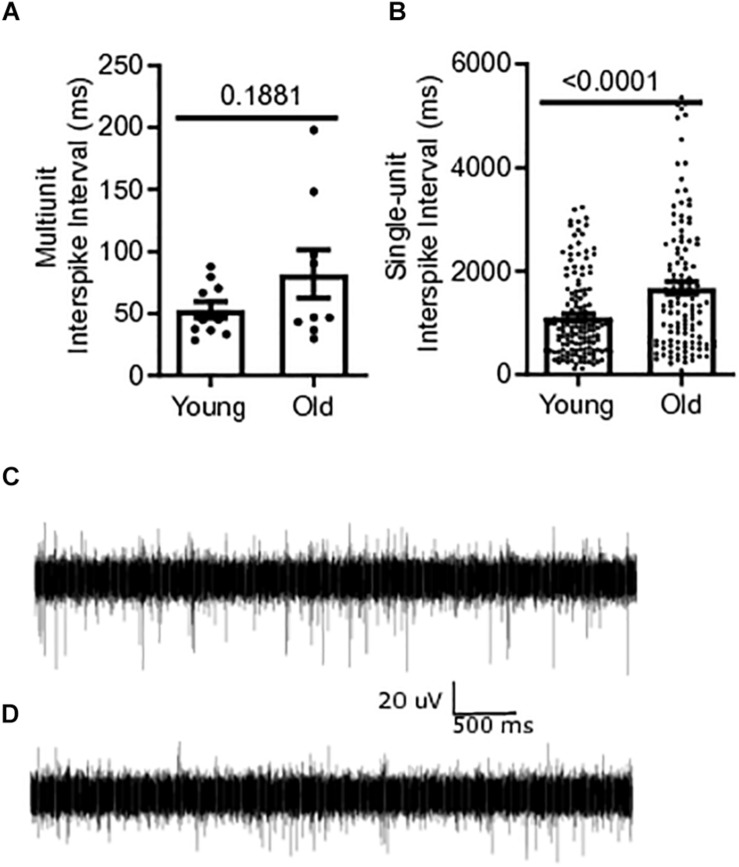
Mesenteric single-unit afferent sensory signaling is decreased in aged mice. **(A)** Multi-unit mesenteric afferent firing from the mesenteric nerve in old mice compared to young mice (*N* = 10 mice). **(B)** Single-unit mesenteric afferent firing from the mesenteric nerve had longer interspike intervals, indicating decreased afferent firing in old mice (*N* = 121 and 149 fibers). **(C)** Representative traces of mesenteric afferent firing for young mice, and **(D)** mesenteric afferent firing for old mice. Data represented as mean with SEM, *t*-test unpaired, two-tailed, with Welch’s correction.

Baseline multiunit mesenteric nerve afferent firing in old (*N* = 9) or young mice (*N* = 10) was measured to determine the effect of aging on afferent discharge, *in vitro*. Afferent firing was measured as mean interspike intervals, in which a decrease in the mean interval between spikes indicates an increase in the firing frequency of the fiber. The multiunit interspike interval of 81.9 ± 19.4 ms for old mice was 55% longer than that for young mice (52.9 ± 6.72 ms) but did not reach statistical significance (*P* = 0.1881) (Figure 3A). Single-unit firing from individual afferent fibers in the mesenteric nerve bundle was identified from multi-unit recordings using DataView as described in the section “Materials and Methods” (Heitler, 2007). The mean interspike interval from all single-unit fibers was 51% longer in old mice, 1676 ± 117.8 ms [*N* = 9(121)], compared to young mice, 1111 ± 63.36 ms [*N* = 10(149)], (*P* < 0.0001) (Figure 3B). A representative trace of multi-unit afferent firing from a young mouse is shown in Figure 3C. Figure 3D shows the same obtained using an old mouse.

### Single Vagal Fiber Firing Rate Is Decreased in Aged Mice but Can Be Rescued by Squalamine

**FIGURE 4:**
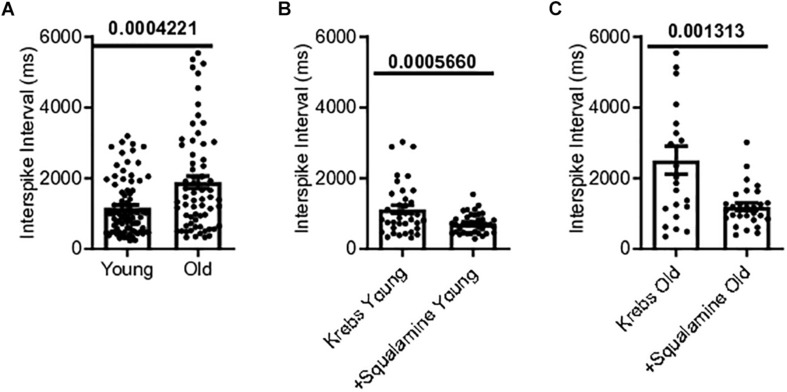
Single vagal afferent firing was reduced in aged mice but could be rescued. **(A)** Mean interval between spikes of vagal afferents were longer in aged mice, indicating a reduction in vagal afferent firing rate in the aged mice (*N* = 148 fibers). **(B)** Intraluminal squalamine (10 μM) reduced the mean interval between vagal spikes in young mice (*N* = 38 fibers). **(C)** Intraluminal squalamine (10 μM) reduced the mean interval between vagal spikes in aged mice, increasing firing frequency toward that of the young controls (*N* = 22 fibers). Data represented as mean with SEM, two-tailed *t*-test unpaired, with Welch’s correction and paired, two-tailed *t*-tests.

Single-unit afferents within the multiunit mesenteric nerve bundle recordings were identified as vagal afferents based on their response to CCK, which selectively stimulates vagal fibers (Grundy et al., 1998). Single unit vagal afferent firing rate was assessed from the mesenteric nerve bundle of the jejunum of young and old mice. Mean vagal single unit interspike intervals were 62% longer for old mice, 1886 ± 176.9 ms [*N* = 10(65)], compared to young mice, 1166 ± 86.22 ms [*N* = 9(83)], (*p* = 0.0004) (Figure 4A).

The ability of squalamine to stimulate vagal fibers was first tested in young mice. Squalamine (10 μM) decreased the mean intervals between vagal spikes by 36% from 1118 ± 117 ms to 718 ± 45.2 ms in young mice [*N* = 3(38), *p* = 0.0006)] (Figure 4B). We then tested whether squalamine could increase the vagal firing rate recorded from the *in vitro* mesenteric nerve preparation taken from old mice. For old mice intraluminal squalamine (10 μM) decreased the mean interval between vagal spikes by 56% from 2509 ± 396 ms to 1093 ± 105 ms [*N* = 4(22), *p* = 0.0013] (Figure 4C). Thus, the reduction in vagal afferent firing for old mice was at least partially reversed by intraluminal squalamine, and squalamine increased vagal firing rate in old mice more strongly than in young mice, 56 vs. 36% increases over the resting firing rates recorded with only Krebs in the lumen.

### Onset to Peak Response of Squalamine Is Longer in Old Mice

**FIGURE 5:**
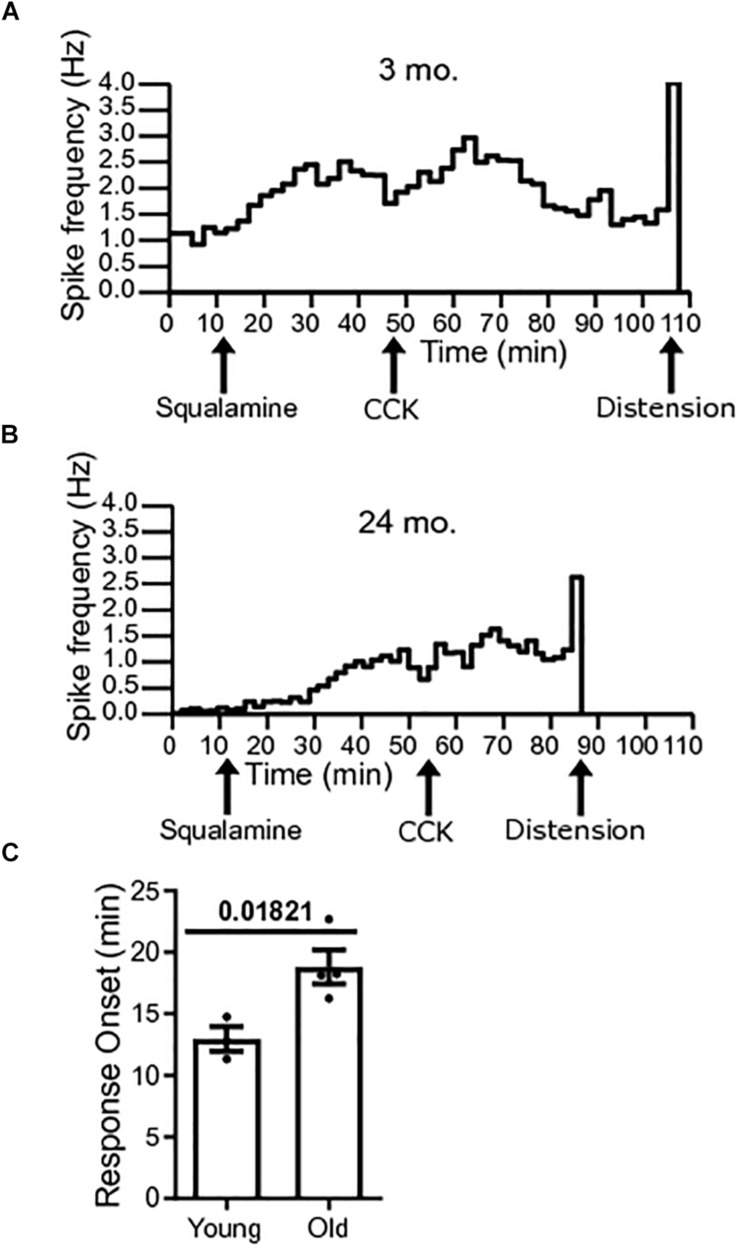
Response onset to squalamine is longer in old mice. **(A)** Spike frequency histograms show changes in spike frequency over the course of the experiment. Onset to peak squalamine response in vagal afferent fibers in a young mouse after addition of squalamine at 12 min. **(B)** Response onset to squalamine in vagal afferent fibers in an old mouse after addition of squalamine. **(C)** Response onset to squalamine on vagal afferent firing rate was longer in old mice [*N* = 4(22)] compared to young mice [*N* = 3(38)]. Data represented as mean with SEM, *t*-test unpaired, with Welch’s correction, two-tailed.

The onset to peak response of squalamine was measured in single-unit vagal afferents of young mice [*N* = 3(38)] and old mice [*N* = 4(22)]. Onset to peak response was measured from the time of addition of squalamine to the lumen to the time that squalamine increased vagal firing rate to peak. The onset to peak response following luminal addition of squalamine was significantly longer for old mice (*P* = 0.0182) (Figure 5A). The onset to peak response in young mice was 12.95 ± 0.99 min compared to 18.82 ± 1.37 min in old mice. Representative frequency histograms over the course of the experiment demonstrate the squalamine onset to peak response to increase vagal afferent firing between young and old mice (Figures 5B,C). We hypothesize that vagal afferent firing is stimulated via an intramural sensory synapse between intrinsic primary afferent neurons (IPANs) of the enteric nervous system and vagal afferent endings (Perez-Burgos et al., 2014). A longer onset to peak response in aged mice would be consistent with a decrease in the excitability of these IPANs in old mice and is discussed later.

## Discussion

In the present study, we used *in vitro* preparations to measure male CD1 mouse colon motility and single unit vagal afferent spike firing rates from the jejunal mesenteric nerve. We showed that motility and spike firing are reduced for old compared to young mice. We also demonstrated that acute intraluminal application of squalamine could partially reverse the effects of old age on motility and constitutive vagal single unit firing rates. The stimulatory effect of squalamine on vagal firing rates had a longer onset latency to peak response in old compared to young mice.

Constipation and decreased gastrointestinal motility disproportionately affect old humans and animal models (Higgins and Johanson, 2004; De Giorgio et al., 2015; Ranson and Saffrey, 2015). For example, aged 2-year-old mice had reduced total fecal output compared to their younger 3-month-old counterparts (Patel et al., 2012); in particular, there was a decrease in velocity of epoxy coated pellet movement in the colon and an increase in impaction (Patel et al., 2012). Despite the large range of laxatives including prokinetics and secretagogues that are available, a significant number of constipated patients are dissatisfied with treatment results (De Giorgio et al., 2015; Sbahi and Cash, 2015). The present work is the first complete report, except for a pilot data abstract in a different mouse model (Kunze et al., 2014), showing that squalamine can partially reverse the old age-related decrease in MMCs velocity in otherwise healthy mice.

Squalamine is a cationic amphipathic sterol with broad antibacterial and antiviral properties (Zasloff et al., 2011) that was first isolated from tissues of the dogfish shark *Squalus acanthias* (Moore et al., 1993). As well as having broad-spectrum anti-microbial activity, squalamine may have potential for treating a variety of diseases such as cancers, age-related macular degeneration and obesity (Brunel et al., 2005). Squalamine has also been shown to restore motility in a *C. elegans* model of Parkinson’s disease (Perni et al., 2017). We have previously reported in abstract form the results of a pilot study showing that the firing rates of vagal afferent fibers are decreased in old compared to young mice and that squalamine increases this function (West et al., 2018). Our present study shows that squalamine can restore colonic motility and vagal afferent firing rates back toward young controls, following a decrease in function with age.

The vagus nerve exhibits anatomic degenerative changes in older animals displaying swollen varicosities (Phillips and Powley, 2007), yet there has so far been little evidence of a reduction in vagal firing in old age. Intestinal mesenteric nerve discharge has been reported to be decreased for old compared to young humans (Yu et al., 2016) and mice (Keating et al., 2015). However, the intestinal mesenteric nerve is a mixed nerve and it is not clear whether the reduction recorded was from vagal, spinal or other fibers. Heart rate variability indices obtained from power spectral density and time domain analysis showed a significant decrease in vagal activity in the elderly (68–85 years) compared to matched young (21–34 years) adults. There was no indication in this study whether afferent, efferent or both types of vagal fibers innervating the heart were involved (Junior and Oliveira, 2017). Of the vagal fibers innervating the gastrointestinal tract we have selected those activated by CCK. We believe our present results are the first to demonstrate reduced constitutive afferent vagal discharge for old compared to young animals. Although the *in vitro* vagal nerve recording methodology is commonly employed, a potential limitation could be that the full scope of sensory signals available *in vivo* may not be available *in vitro*.

Although the vagus can modulate small and large intestinal motility (Collman et al., 1983, 1984; Raybould and Tache, 1988; Gustafsson and Delbro, 1994; Tong et al., 2010), it is not known whether or to what extent reduced vagal firing rates contribute to a decrease in propulsive peristalsis in old mice *in vivo*. Chronic extrinsic denervation of the intestine allowing extrinsic nerve fibers to fully degenerate does not abolish or alter peristalsis reflexes (Furness et al., 1995) further emphasizing that MMCs are generated by the ENS. On the other hand, propulsive peristalsis directly reflects intrinsic primary afferent neuron (IPAN) functioning (Howe et al., 2006) and the same IPANs transmit to vagal afferent endings (Perez-Burgos et al., 2014) suggesting that compromised IPAN functioning may be an important determinant in reduced propulsion and vagal firing in old age. Therefore, it may be more likely that reduced colonic motility and reduced vagal afferent firing are not completely causally linked to each other. Because squalamine stimulates colonic motility in the absence of extrinsic innervation, it is likely that squalamine stimulated IPAN firing and that this may produce improvements in both the aged colon motility and vagal afferent firing.

The increased onset latency to peak response of squalamine on afferent vagal firing in tissue taken from old compared to young mice may be interpreted by the effects of old age on the enteric nervous system. Ninety to 95% of sensory neuron processes innervating the intestinal epithelium arise from the ENS, with the rest originating from neurons whose somata are located outside the intestine (Keast et al., 1984; Ekblad et al., 1987). In agreement with this anatomical data, is our recent discovery that more than two thirds of vagal afferent signals evoked by a luminal commensal bacteria is relayed to the vagus via the enteric neurons (Perez-Burgos et al., 2014). Neuroactive luminal molecules first excite juxtaepithelial neurites belonging to IPANs whose cell bodies are located within the ENS. The excited IPANs release acetylcholine and perhaps other neurotransmitters to activate vagal IGLEs which closely surround and abut the IPANs (Berthoud et al., 1997; Perez-Burgos et al., 2014). The IPAN to IGLE nicotinic sensory synapse we have described (Perez-Burgos et al., 2014), is perfectly positioned to act as a gatekeeper to regulate gut to brain signaling. Accordingly, the amount of information transmitted to the brain via the vagus would be markedly influenced by whether IPANs are refractory or readily responsive to luminal stimuli, and by the density of IPAN sensory innervation of the epithelium. Given the important role of enteric IPANs in gut to brain signaling (Perez-Burgos et al., 2014), the vulnerability of the ENS to old age in terms of numbers and degeneration could have a significant impact on the amount, quality and latency of signals reaching the brain from the gut. Thus, even if the numbers of vagal afferent fibers are not appreciably reduced in old age there could be decreased and delayed vagal afferent responses to luminal stimuli.

Functional GI disorders are often comorbid with mood and anxiety disorders (Ballou et al., 2019). In a study of 54 constipated patients with motility disorders, 22.2% showed depression using the Hospital Anxiety and Depression Scale (HADS) and 31.5% on the Mini-International Neuropsychiatric Interview (MINI) (Hosseinzadeh et al., 2011). As previously mentioned, constipation incidence and prevalence increases with age (Higgins and Johanson, 2004; De Giorgio et al., 2015; Ranson and Saffrey, 2015) and depression is common in old age (Beekman et al., 1999; Djernes, 2006; McCall and Kintziger, 2013). Clinically relevant depressive symptoms may, for example, reach a prevalence of up to 49% in institutionalized elderly Caucasians (Djernes, 2006). Indeed in a cross-sectional study between psychiatric diagnoses and constipation, inpatients older than 60 years had a significantly increased risk of constipation (odds ratios 3.38–6.52) (Jessurun et al., 2016). Increased depression in old age may be related, at least in part, to alterations in vagus nerve activity (Forsythe et al., 2014) and successful treatment of depression may also relieve the associated constipation. Vagal nerve stimulation is FDA approved, and shows promise as an essential or adjunct antidepressant treatment in humans (Carreno and Frazer, 2017; Johnson and Wilson, 2018). Indeed, a 5-year observational follow up of vagal nerve stimulation in depression has shown very promising results (Aaronson et al., 2017).

Activation of the visceral afferent vagus appears to have antidepressant behavioral and mood-altering effects in mice. Indeed, the antidepressant effects of certain neuroactive microbes, which increase discharge frequency in mesenteric vagal afferents (Perez-Burgos et al., 2013), depend on the presence of an intact vagal nerve since subdiaphragmatic vagotomy abrogated both the antidepressant effects and regional changes in GABA receptor expression in the brain (Bravo et al., 2011).

Our results show that vagal afferent firing is reduced in old age, but these changes are not permanent regardless of causation since these effects can be restored to within range of the young mouse controls. Future studies should seek to evaluate the effect of reduced vagal afferent firing in old age on depression and also GI function as a whole. Additionally, experiments should be repeated *in vivo* where possible to test for translatability of the *in vitro* results. The marked improvement induced by squalamine in the disordered motility of aged mice may have a translatable clinical use in treating old age-related constipation and awaits clinical testing.

## Data Availability

The datasets generated for this study are available on request to the corresponding author.

## Ethics Statement

This study was approved by and conducted in accordance with the Animal Research Ethics Board (AREB) of McMaster University (permit 16-08-30). Experiments were performed *in vitro* following cervical dislocation.

## Author Contributions

CW, JA, and SF performed the colonic motility experiments and analyzed the data. CW performed the mesenteric nerve experiments and analyzed the data. Y-KM and AS provided the technical support for the experiments. CW and WK prepared the figures, and wrote and reviewed the manuscript. CW, AS, and WK contributed to the conception, design, facilitation, and supervision of the study. All authors read and approved the manuscript.

## Conflict of Interest Statement

WK is member of the scientific advisory board of Enterin Inc., but has received no personal remuneration from them. Enterin Inc., was not involved in any aspect of the design or conduct of this study, including the preparation of the manuscript. The remaining authors declare that the research was conducted in the absence of any commercial or financial relationships that could be construed as a potential conflict of interest.
